# Immunoglobulin G4‐Related Inflammatory Pseudotumor With Cystic Features Mimicking Renal Cancer

**DOI:** 10.1002/iju5.70074

**Published:** 2025-07-27

**Authors:** Yuki Tanaka, Takahisa Yamashita, Kazuki Yokota, Kenta Fujii, Sachi Kitayama, Shoichi Nagamoto, Hideki Takeshita, Yohei Okada, Satoru Kawakami, Akihiro Yano

**Affiliations:** ^1^ Department of Urology Saitama Medical Center, Saitama Medical University Kawagoe Japan; ^2^ Department of Pathology Saitama Medical Center, Saitama Medical University Kawagoe Japan

**Keywords:** cysts, immunoglobulin G4‐related disease, inflammatory pseudotumor, kidney diseases, kidney neoplasms

## Abstract

**Introduction:**

Immunoglobulin G4‐related disease is a systemic fibroinflammatory disorder that affects the kidney, presenting as an immunoglobulin G4‐related inflammatory pseudotumor. These renal inflammatory pseudotumors are usually solid, and cystic presentations have not been previously described.

**Case Presentation:**

We report a 77‐year‐old man who presented with a Bosniak category III renal cystic mass and periaortic fibrous thickening. Increased serum immunoglobulin G4 levels indicated immunoglobulin G4‐related disease; however, a malignant process could not be definitively ruled out. The patient underwent robot‐assisted radical nephrectomy, and histopathologic assessment confirmed a cystic immunoglobulin G4‐related inflammatory pseudotumor.

**Conclusion:**

This is the first report of a renal immunoglobulin G4‐related inflammatory pseudotumor with cystic features. Increased knowledge of immunoglobulin G4‐related disease as a potential cause of cystic renal lesions, particularly in patients with systemic findings, may improve preoperative diagnosis.

**Summary**

- We present the first described case of a renal immunoglobulin G4 (IgG4)‐related inflammatory pseudotumor with cystic characteristics.
- Preoperative consideration of IgG4‐related disease in cystic renal lesion assessment may improve diagnostic precision and minimize unnecessary surgical procedures.

## 1 Introduction

Immunoglobulin G4 (IgG4)‐related disease is a systemic fibroinflammatory condition defined by increased serum IgG4 levels and characteristic histopathological findings, remarkably dense lymphoplasmacytic infiltrates, storiform fibrosis, and obliterative phlebitis [1, 2]. IgG4‐related kidney disease, representing renal involvement in IgG4‐related disease, commonly presents as tubulointerstitial nephritis but can also manifest as a tumorlike lesion—IgG4‐related inflammatory pseudotumor (IPT) [1, 2, 3]. These tumorlike lesions pose a diagnostic dilemma because their radiographic appearance can imitate renal malignancies, such as renal cell carcinoma (RCC) and renal pelvis urothelial carcinoma [4, 5, 6, 7, 8, 9, 10, 11, 12, 13].

Renal IgG4‐related IPT has been almost exclusively reported as a solid tumor; however, cystic features have not been previously documented [4, 5, 6, 7, 10, 11, 12]. We report the first case of renal IgG4‐related IPT exhibiting cystic features, initially suspected to be a cystic renal neoplasm. This case highlights the need to include IgG4‐related IPT in the differential diagnosis of renal tumors, including those with cystic characteristics.

## 2 Case Presentation

**FIGURE 1:**
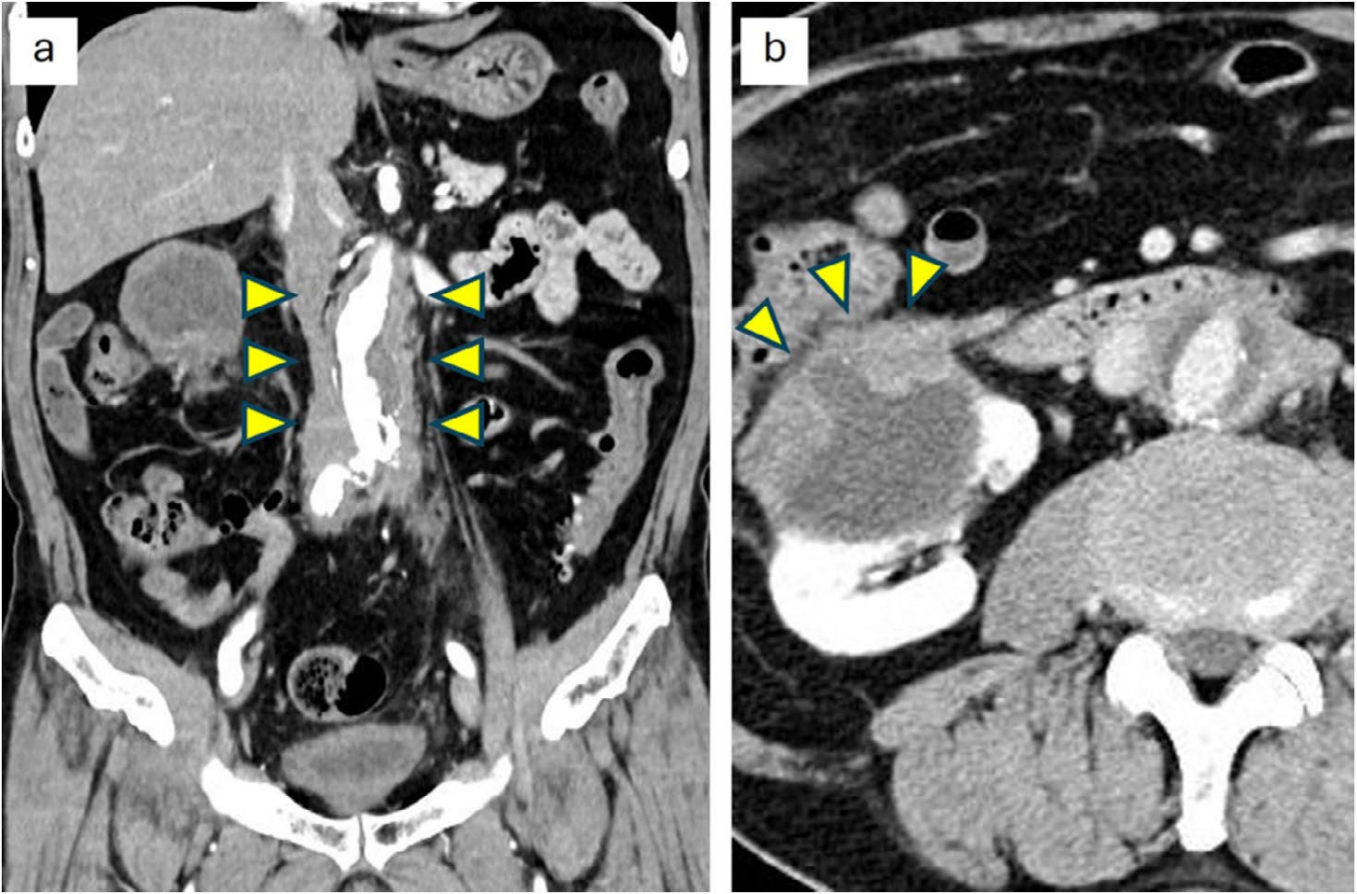
Contrast‐enhanced computed tomography imaging of a right renal cystic mass. Arrows represent the lesion. (a) Abdominal aorta atherosclerosis with periaortic fibrous thickening. (b) A 6.7‐cm cystic mass in the right kidney, characterized by thickened walls and mild enhancement, is classified as Bosniak category III.

**FIGURE 2:**
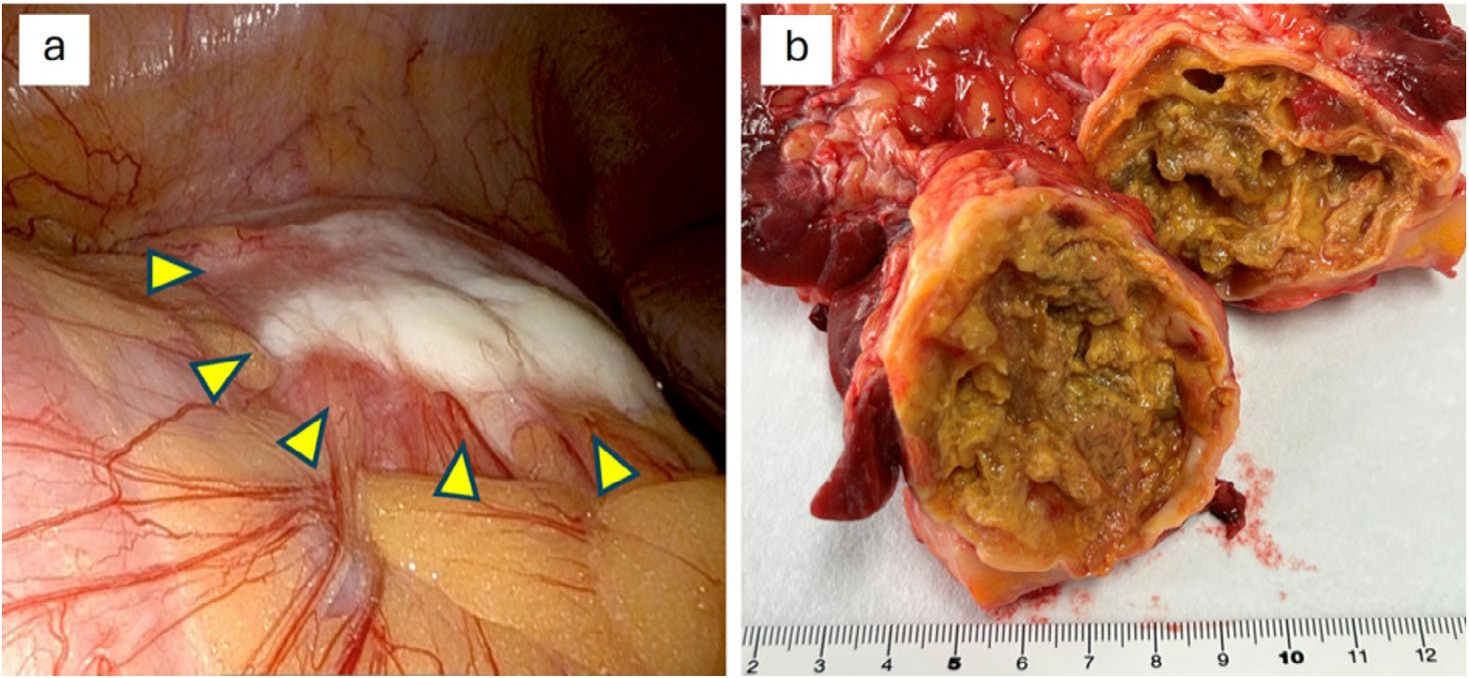
Gross appearance of the right renal cystic mass. Arrows indicate the lesion. (a) Anterior fascia of the cystic lesion is fibrotic, whitish, and covered with fibrin exudation. In the image, the right side is cranial, whereas the left side is caudal. (b) The resected specimen exhibits a renal cyst containing a yellowish solid component.

A 77‐year‐old man visited an outside hospital due to low‐extremity edema. A suspected renal tumor, identified on imaging, resulted in his referral to our hospital for further assessment. Contrast‐enhanced computed tomography imaging revealed abdominal aorta atherosclerosis and periaortic fibrous thickening (Figure 1a), as well as a 6.7‐cm right renal cystic lesion with thickened walls and mild enhancement, consistent with Bosniak category III (Figure 1b). Laboratory assessment revealed an increased IgG4 level of 250 mg/dL (reference range: ≤ 135 mg/dL) and a normal C‐reactive protein level of 0.04 mg/dL. Consequently, the differential diagnosis included both RCC and IgG4‐related diseases. Considering the potential risk of tumor seeding, a renal biopsy was not performed, and the patient underwent robot‐assisted radical nephrectomy. Intraoperative findings included a white and discolored anterior fascia of the cystic lesion (Figure 2a), along with marked adhesions to the ascending colon and duodenum, which complicated the dissection. The periaortic lesion, identified to be separated from the renal tumor, was left in situ. The total surgical time was 7 h and 14 min; the robotic console time was 6 h and 11 min, and the estimated blood loss was 25 mL. After discharge, the patient was observed but not prescribed steroid therapy.

**FIGURE 3:**
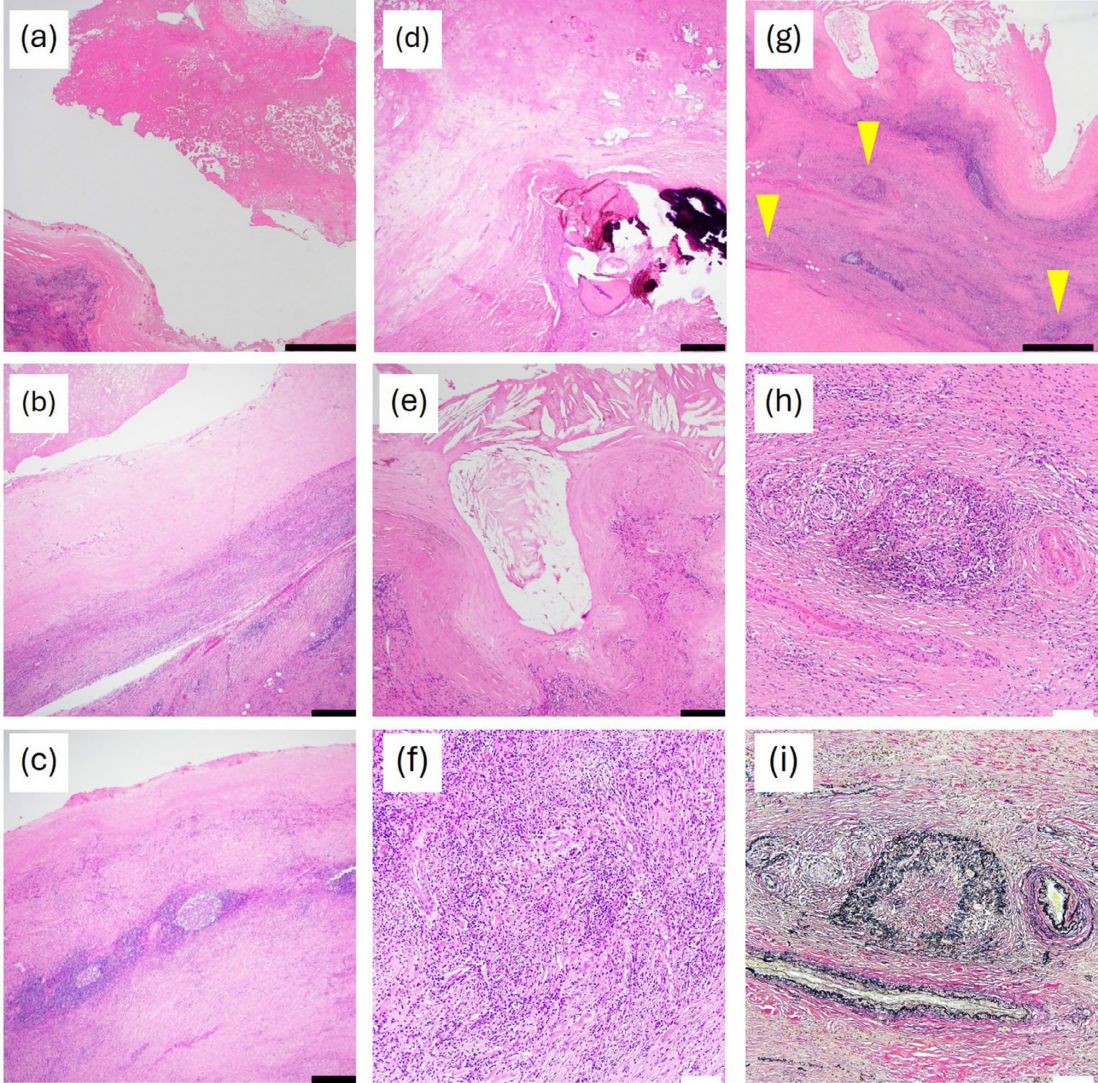
Histological analysis of the renal cyst wall. (a) Fibrious material is observed within the cyst lumen. (b) Fibrous tissue exhibiting hyaline degeneration in a portion of the cyst wall. (c) Secondary lymphoid follicle formation within the cyst wall. (d) Calcification within the cyst wall. (e) Cholesterol clefts in a part of the cyst wall. (f) Storiform fibrosis with lymphoplasmacytic infiltration within the cyst wall and adjacent tissue. (g) Obliterative phlebitis (yellow arrowheads) within the cyst wall and surrounding tissue. (h, i) High‐magnification views of the lesion (g), stained with hematoxylin and eosin (h) and Elastica van Gieson (i), respectively. Scale bars: Black = 1 mm, white = 100 μm.

**FIGURE 4:**
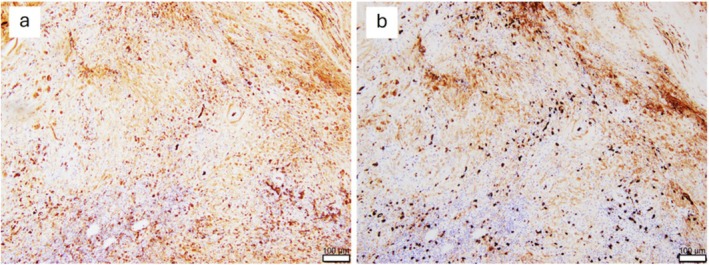
Immunohistochemical findings in the right renal cyst wall and surrounding tissue. (a) IgG staining. (b) IgG4 staining. The ratio of IgG4‐positive to IgG‐positive cells is > 50%. Sixty to seventy IgG4‐positive cells per high‐power field are observed.

A gross examination of the resected specimen revealed a renal cyst containing a yellowish solid component (Figure 2b). Histopathological analysis revealed fibrinous material within the cyst, hyalinized changes, secondary lymphoid follicle formation, calcifications, and cholesterol clefts within the cyst wall (Figure 3a–e). The inflammatory infiltrate consisted predominantly of lymphocytes and plasma cells, with scattered eosinophils. Neutrophilic infiltration was inconspicuous. Further, storiform fibrosis, with lymphoplasmacytic infiltration and obliterative phlebitis, was observed within the cyst wall and surrounding tissues (Figure 3f–i). Immunohistochemical staining revealed CD138‐positive plasma cell infiltration, and the IgG4/IgG‐positive cell ratio was > 50%, with 60–70 IgG4‐positive cells per high‐power field observed in the cyst wall and surrounding tissue (Figure 4a,b). These histopathologic and immunohistochemical results confirmed IgG4‐related IPT diagnosis.

## 3 Discussion

This is the first documented case of a renal IgG4‐related IPT presenting with cystic features, indicating the diagnostic challenge of distinguishing it from malignant neoplasms. Previous reports of renal IPT have almost uniformly described solid masses; however, cystic lesions have not been previously reported in this organ. Differentiating cystic IgG4‐related IPT from malignant cystic renal tumors is clinically difficult. Malignant cystic tumors typically exhibit enhancing mural nodules or irregular wall thickening on contrast‐enhanced computed tomography [14]. In contrast, IgG4‐related IPTs may present as low‐attenuation lesions without clear enhancement and can be accompanied by extrarenal fibroinflammatory lesions, such as periaortic or retroperitoneal fibrosis [3, 7, 10]. Further, increased serum IgG4 levels, a high ratio of IgG4‐positive to IgG‐positive plasma cells, storiform fibrosis, and obliterative phlebitis on histopathological assessment support the diagnosis of IgG4‐related disease [1]. However, obtaining adequate tissue samples in cystic lesions through biopsy is often challenging, and the potential risk of tumor seeding further limits preoperative diagnosis. In our patient, imaging studies indicated a thickened and irregular cystic lesion, classified as Bosniak category III. This designation typically prompts surgical intervention due to its inherent malignant potential [14]. Remarkably, this patient presented with periaortic fibrous thickening and increased serum IgG4 levels, both of which are IgG4‐related disease characteristics. These additional clinical and laboratory findings could have indicated IgG4‐related IPT diagnosis preoperatively, thereby potentially resulting in a more accurate diagnosis.

A total of 49 cases of renal IPT were reported between 1999 and 2022, with the majority initially suspected to represent malignancy and subsequently treated surgically. Of these, 7 (14%) were classified as IgG4‐related, whereas the remaining 42 were considered non‐IgG4‐related. The non‐IgG4‐related cases were associated with a wide variety of underlying conditions or etiologies, such as Behçet's disease, infections (e.g., Histoplasma capsulatum and Epstein–Barr virus), systemic inflammatory diseases (e.g., rheumatoid arthritis, granulomatosis with polyangiitis, and gout), and other conditions reported in individual cases (e.g., chronic pachymeningitis, primary amenorrhea, chronic hepatitis B, Raynaud's phenomenon, ipsilateral perinephric, and periureteric fibrosis) [15]. All seven renal IgG4‐related IPT cases described in the literature presented with solid masses [4, 5, 6, 7, 10, 11, 12]. Cystic IgG4‐IPT has not been previously reported in the kidney; however, it has been documented in other organs such as the lungs, thymus, and ovaries [16, 17, 18, 19]. The exact mechanism underlying cyst formation in IgG4‐related disease remains unclear; however, chronic inflammation during disease progression is indicated to contribute to cystic changes in pulmonary cases [16, 17]. In the present case, the presence of extensive lymphoplasmacytic infiltration, storiform fibrosis surrounding the cyst, and histological features, such as cholesterol clefts and hyalinization, may reflect long‐standing inflammation and tissue remodeling. While speculative, local ischemia due to obliterative phlebitis could have played a role in tissue alteration. Further research, including the study of additional cases and detailed pathological analyses, is warranted to understand the responsible mechanisms for cyst formation in IgG4‐related diseases. Appropriate clinical management strategies must also be considered in addition to understanding the pathogenesis. An IgG4‐related renal tumor should be suspected if extrarenal IgG4‐related disease is present, even if the tumor is cystic. Surgical intervention was unavoidable in the present case due to the presence of a Bosniak category III cystic lesion and the inability to exclude malignancy preoperatively. However, careful observation, particularly when supported by systemic findings, may be considered an alternative to immediate surgery in cases where sufficient tissue sampling is possible, and IgG4‐related disease can be confirmed through biopsy. Further accumulation of such cases may help improve diagnostic strategies and avoid unnecessary nephrectomy.

## Consent

Written informed consent was obtained from the patient.

## Conflicts of Interest

The authors declare no conflicts of interest.

## Data Availability

The authors have nothing to report.
